# Durable complete response to PD-1 inhibitor in vesical calculus-associated squamous cell carcinoma: a case report

**DOI:** 10.3389/fimmu.2025.1688443

**Published:** 2025-11-21

**Authors:** Xingwang Dong, Xiaobao Fu, Hanghao Zhang, Yingjun Ma, Cheng Wang, Ye Li, Su Zhang

**Affiliations:** 1 Department of Urology, Gannan Tibetan Autonomous Prefecture People’s Hospital, Hezuo, China; 2 Department of Urology, Lanzhou University Second Hospital, Lanzhou, China; 3 Cuiying Honors College, Lanzhou University, Lanzhou, China; 4 Department of Pathology, West China Second University Hospital, Sichuan University, Chengdu, China

**Keywords:** bladder, squamous cell carcinoma, vesical calculus, PD-1 inhibitor, immunotherapy

## Abstract

**Background:**

Bladder squamous cell carcinoma (SCC) is a rare histological subtype of bladder cancer (1%–5% of cases), with radical cystectomy as the primary recommended treatment. However, evidence for neoadjuvant/adjuvant chemotherapy or immunotherapy remains limited, especially for calculus-associated SCC.

**Case presentation:**

A 45-year-old male with vesical calculus-associated bladder SCC refused surgery and radiotherapy, and discontinued gemcitabine-cisplatin chemotherapy due to severe toxicity. He received tislelizumab (programmed death-1/PD-1 inhibitor, 200 mg q3w) and achieved a complete response (CR) after 8 cycles. Suprapubic cystolithotomy was safely performed during immunotherapy, and no tumor recurrence was observed for >24 months. Throughout the entire immunotherapy course, no severe immune-related adverse events occurred.

**Conclusion:**

Tislelizumab monotherapy may be a viable option for surgery-ineligible or chemotherapy-intolerant calculus-associated bladder SCC. Definitive stone removal under immunological tumor control is feasible, supporting further exploration of PD-1 inhibitors in this rare subtype.

## 1 Introduction

Bladder cancer represents one of the most prevalent urinary tract malignancies, with urothelial carcinoma (UC) constituting the predominant histological subtype (>90% of cases). In contrast, squamous cell carcinoma (SCC) is a rare variant accounting for only 1%-5% of bladder cancer diagnoses (1). Bladder SCC is categorized into schistosomal and non-schistosomal subtypes. The schistosomal variant arises from Schistosoma haematobium egg deposition in the bladder mucosa, triggering chronic inflammation, progressive tissue damage, and subsequent malignant transformation. This subtype exhibits distinct geographical distribution and is endemic primarily in Middle Eastern regions. Conversely, non-schistosomal SCC typically develops secondary to persistent bladder irritation by chronic conditions such as vesical calculi, prolonged catheterization, or urinary tract obstruction. While radical cystectomy remains the cornerstone for localized disease, optimal management for inoperable patients remains undefined, with limited evidence for systemic therapies (2). Herein, we present a clinically informative case: a 45-year-old male with calculus-induced SCC achieving sustained complete response (>27 months) on PD-1 inhibitor tislelizumab (BeiGene) monotherapy after declining surgery and failing chemotherapy.

## 2 Case presentation

**Figure 1:**
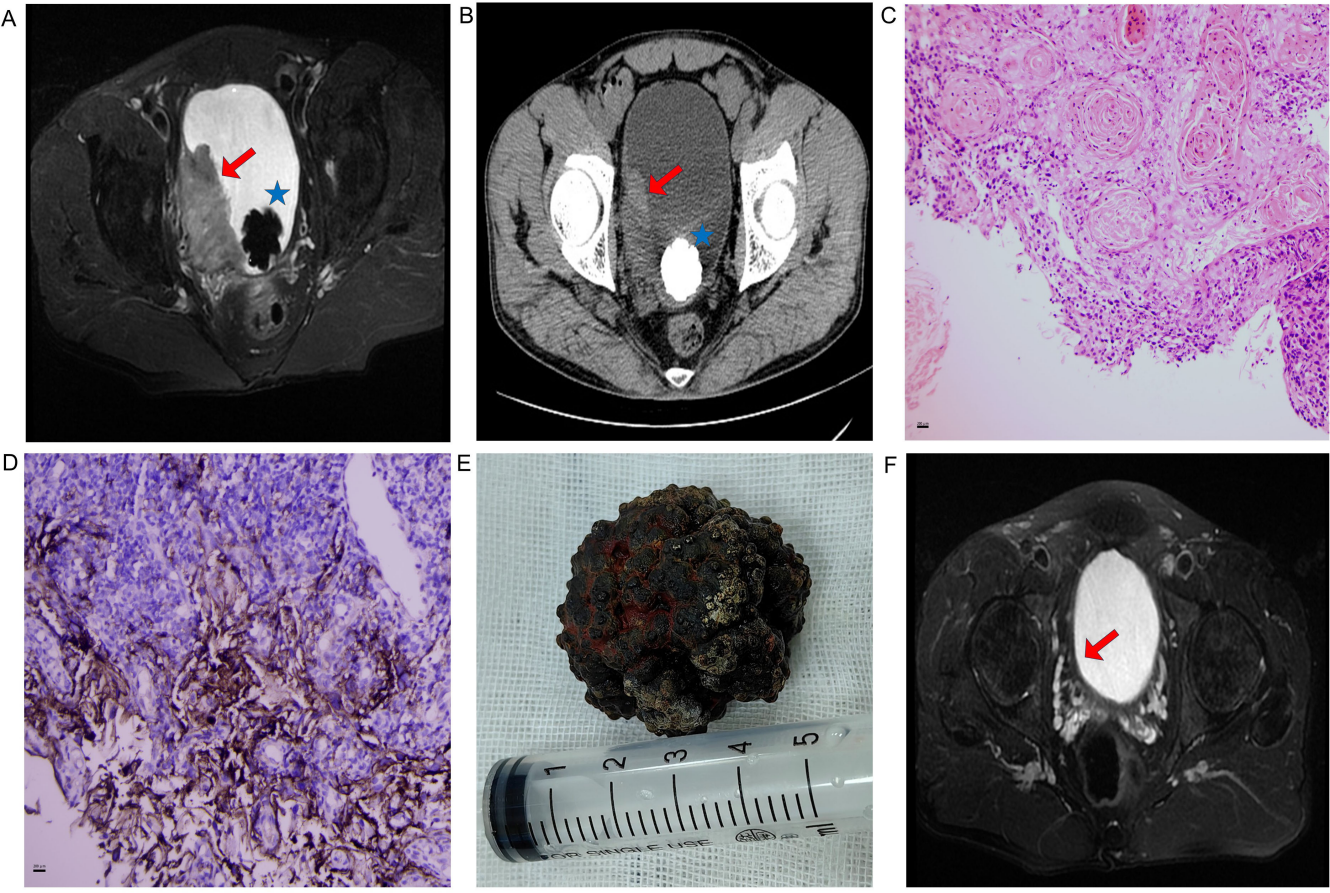
**(A)** Pre-treatment pelvic MRI (axial T2-weighted sequence): Red arrow indicates a 5.8×3 cm heterogeneous mass on the right lateral bladder wall; blue asterisk indicates an associated vesical calculus. **(B)** Pre-treatment pelvic CT: Red arrow indicates a 5.8×3 cm heterogeneous mass on the right lateral bladder wall; blue asterisk indicates an associated vesical calculus. **(C)** Histopathological confirmation (H&E staining, ×200) revealing nests of squamous carcinoma cells with keratin pearls and stromal invasion. **(D)** Immunohistochemical profiles (×200): PD-L1 (22C3 pharmDx, CPS >1). Brown staining indicates positive tumor cells. **(E)** Surgical specimen: calculus retrieved via open cystolithotomy. **(F)** Post-immunotherapy MRI (axial T2-weighted sequence) showing complete resolution of the tumor after 12 cycles of tislelizumab. Red arrow indicates the former tumor bed, with no evidence of residual tumor.

A 45-year-old male presented to the Urology Department in January 2023 with intermittent gross hematuria accompanied by lower urinary tract symptoms (dysuria, urinary frequency, urgency, and voiding difficulty). The patient denied constitutional symptoms such as nausea or vomiting. Physical examination revealed no abnormalities; the patient had an Eastern Cooperative Oncology Group (ECOG) performance status of 1 at baseline and no comorbidities (e.g., hypertension, diabetes, cardiovascular disease). The patient was a long-haul truck driver with a mild smoking history, no alcohol consumption history, and no occupational toxin exposure history. There was no family history of malignant tumors (especially urinary tract malignancies) or genetic disorders. Urinalysis confirmed microscopic hematuria (RBC +++). Complete blood count showed severe anemia (hemoglobin 66 g/L) with otherwise normal parameters. Pelvic MRI and CT revealed a 5.8 × 3 cm mass in the right lateral bladder wall with an associated vesical calculus, without lymphadenopathy (**Figures 1A, B**). Cystoscopic biopsy confirmed invasive squamous cell carcinoma (**Figure 1C**). Immunohistochemical analysis showed diffuse positivity for P40, P63, and CK5/6, with a high Ki-67 proliferation index (70%). Negative staining was observed for GATA3, CK7, and CK20. PD-L1 expression was positive (combined positive score [CPS] >1), assessed using the 22C3 pharmDx assay (**Figure 1D**) (Agilent Technologies, Santa Clara, CA, USA).

Following diagnostic confirmation, the patient and family received comprehensive counseling regarding disease status and proposed treatment options. They declined both surgical intervention and radiotherapy. After one cycle of gemcitabine-cisplatin chemotherapy, treatment was discontinued due to severe adverse effects: the patient developed severe nausea and vomiting (which remained uncontrolled even with standard antiemetic regimens including ondansetron and aprepitant), along with persistent leukopenia (white blood cell count stayed below 2×10^9^/L) and thrombocytopenia (platelet count remained below 40×10^9^/L). Despite supportive care, the patient still experienced persistent fatigue and gastrointestinal discomfort, and thus explicitly refused to continue with chemotherapy. Given positive PD-L1 expression (CPS > 1), tislelizumab (200 mg every 3 weeks) was maintained as consolidation therapy after obtaining written informed consent from the patient. Work-related constraints limited adherence to the standard protocol: the patient, a long-haul truck driver, needed to conduct prolonged cross-provincial transportation tasks, which prevented him from returning to the hospital on time. Follow-up assessments revealed near-complete resolution of hematuria after the third cycle of immunotherapy. Subsequently, significant tumor regression was observed after the fourth cycle—coinciding with improved treatment adherence from the patient. At this point, the patient continued to experience intractable voiding pain, which necessitated vesical calculus extraction. Given the calculus size and potential tumor seeding risks associated with prolonged transurethral lithotripsy, open cystolithotomy was performed (**Figure 1E**). Further imaging after the fifth cycle confirmed substantial tumor response, with >90% tumor volume reduction on pelvic MRI. Ultimately, a complete response was achieved after the eighth cycle of immunotherapy. By Month 8 (concurrent with the fourth to fifth cycles of treatment), the patient reported significant quality of life improvement: he had resumed full-time work as a long-haul truck driver without activity limitations.

**Figure 2:**
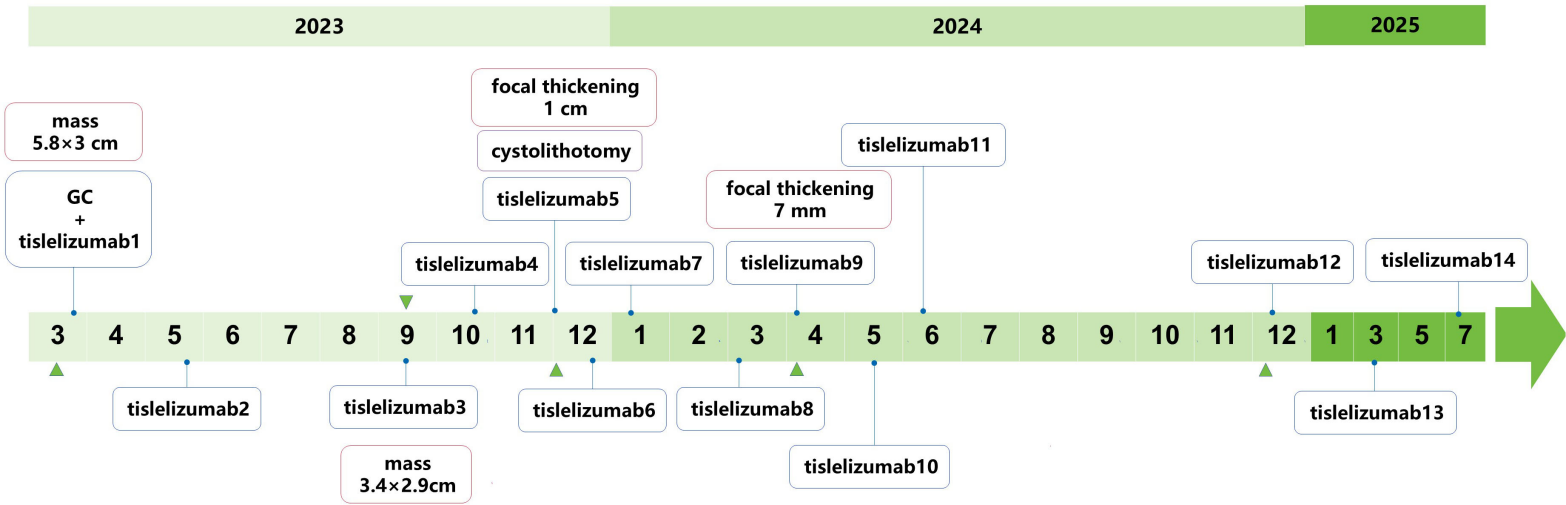
Treatment timeline of PD-L1+ bladder SCC with giant calculus. Blue diamond: Tislelizumab immunotherapy cycles (200 mg q3w); green triangles: Radiological response assessments (pelvic MRI/CT).

By June 2025, the patient had completed 14 intermittent cycles of tislelizumab monotherapy. Notably, the patient’s existing treatment course (14 cycles) deviates from the expected 2-year regimen (planned to be administered once every 3 weeks, totaling approximately 32 cycles), yet tumor control remains excellent. *Additionally, d*uring the 14 cycles of tislelizumab monotherapy, the patient was monitored for immune-related adverse events (irAEs) per institutional guidelines. No grade ≥2 irAEs were observed; the only irAE reported was intermittent thrombocytopenia, with platelet counts consistently remaining above 60×10^9^/L. This thrombocytopenia was effectively improved with oral medications (ampeptide elemente or coffee acid tablets) and did not require dose modification or treatment interruption. Despite this favorable safety profile, given the rarity of calculus-associated bladder SCC and the need for long-term recurrence monitoring, a standardized surveillance protocol was formulated: cystoscopy every 3 months plus pelvic MRI/CT every 3–6 months for the first 2 years, and cystoscopy every 6 months plus annual pelvic MRI/CT from the 3rd year onward. However, the patient failed to adhere well to this plan, primarily due to his occupation as a long-haul truck driver. To mitigate associated risks, the medical team implemented monthly telephone follow-ups (to inquire about urinary symptoms and general condition). Ultimately, all these surveillance assessments showed no evidence of tumor recurrence (**Figure 1F**) and specific treatment timepoints are detailed in **Figure 2**.

## 3 Discussion and conclusions

Bladder SCC primarily manifests with hematuria. Given its rarity, current therapeutic evidence primarily derives from retrospective studies with limited sample sizes, restricting the development of evidence-based clinical guidelines. For localized disease, radical cystectomy is recommended as primary definitive therapy without neoadjuvant approaches. Conversely, no standardized protocols exist for metastatic SCC, though sporadic case reports indicate potential benefit from conventional chemotherapy regimens with radiotherapy (1). Notably, a recent SEER database analysis revealed that chemoradiotherapy offered no survival advantage and may negatively impact outcomes in non-schistosomal bladder SCC. Surgical intervention remains as the sole potentially curative modality for localized disease (3). The treatment landscape for bladder urothelial carcinoma has evolved significantly, with widespread adoption of neoadjuvant chemotherapy and bladder-preserving strategies. Immune checkpoint inhibitors and antibody-drug conjugates have further revolutionized management, overcoming historical limitations of toxicity and therapeutic constraints (4). In contrast, evidence for non-urothelial bladder tumors remains limited. However, emerging data on PD-L1 expression in bladder SCC have provided a potential direction for immunotherapy, offering a breakthrough opportunity to address this unmet need.

PD-L1 positivity rates exceed 60% in both schistosomiasis-associated and non-schistosomal bladder SCC cohorts across geographically distinct populations—significantly higher than the ~20% typically observed in UC (5–7). Our case aligns with a 2019 report by Kao et al. (8). Specifically, Kao et al. described a cisplatin-ineligible patient with non-schistosomal bladder SCC. This patient developed tumor recurrence with distant lymph node metastases following two transurethral resections of bladder tumor (TURBT) and radical cystectomy. Ultimately, the patient achieved a complete response after receiving 13 cycles of pembrolizumab. However, our study has three key distinctions (1): Our patient had calculus-associated bladder SCC—a subtype for which data on immunotherapy efficacy remain limited—whereas Kao et al.’s case involved idiopathic non-schistosomal bladder SCC (2); Our patient underwent definitive vesical calculus removal via suprapubic cystolithotomy during immunotherapy, demonstrating that invasive procedures for comorbid conditions (e.g., vesical calculus) can be safely performed when tumors are under stable immunological control (3); Critically, our patient achieved durable CR without undergoing any tumor-related surgical intervention, whereas Kao et al.’s patient had already received multiple surgical treatments before immunotherapy initiation. Beyond these clinical differences, the PD-1 inhibitor used in our case—tislelizumab—also differs mechanistically from pembrolizumab and nivolumab, which may contribute to the durable efficacy observed herein. Tislelizumab, a humanized IgG4 anti-PD-1 antibody, differs mechanistically from pembrolizumab and nivolumab in two key aspects. First, tislelizumab is the only PD-1 monoclonal antibody with fully successful Fc segment engineering. This modification minimizes its binding to Fcγ receptors on macrophages and abrogates antibody-dependent phagocytosis of activated T cells, whereas pembrolizumab lacks such Fc modification. Second, tislelizumab exhibits a significantly slower dissociation rate from wild-type PD-1 compared with the other two antibodies: it is approximately 100-fold slower than pembrolizumab and 50-fold slower than nivolumab. This “slow-off” property is attributed to its unique binding epitope on PD-1 (9). These two mechanistic differences may explain the favorable efficacy observed in the case reported herein.

To our knowledge, this is the first documented case of tislelizumab monotherapy achieving a durable complete response in calculus-associated bladder SCC without radical cystectomy. While our case demonstrates tislelizumab’s efficacy in patients with calculus-associated bladder SCC who are ineligible for surgery or intolerant to chemotherapy, long-term disease control for this subtype still faces unresolved challenges (1): Late recurrence (≥2 years post-CR) in immunotherapy-responsive urological cancers requires lifelong surveillance (2); Acquired resistance to PD-1 inhibitors may occur via mechanisms such as loss of PD-L1 expression or activation of alternative immune checkpoints, for which salvage therapies (e.g., antibody-drug conjugates) are untested in bladder SCC (3); The optimal maintenance duration is undefined: current guidelines recommend 12–24 months for most solid tumors, but our patient’s durable CR suggests extended therapy may be beneficial for high-risk subtypes (4); No evidence-based salvage strategies exist for recurrence (e.g., antibody-drug conjugates, genomic-profiled agents, salvage cystectomy, or radiotherapy) (5); Genomic profiling (e.g., TMB, MSI-H) could identify additional biomarkers, but such data are scarce in calculus-associated SCC. These gaps highlight the need for etiology-based molecular subtyping of bladder SCC and prospective trials to define optimal therapeutic sequences.

While the above challenges reflect unmet clinical needs for calculus-associated bladder SCC, our study also has inherent limitations that should be considered when interpreting the findings (1): It is a single-case report, which limits the generalizability of our observations to other patients with bladder SCC (e.g., schistosomiasis-associated subtypes or those with different PD-L1 expression levels) (2). We lack long-term follow-up beyond 32 months; while the patient has maintained CR to date, longer surveillance is needed to assess for late recurrence (3). Genomic profiling was not performed, precluding identification of additional response biomarkers. Given these limitations, larger prospective cohort studies or multicenter trials are urgently needed to validate the efficacy of PD-1 inhibitors in calculus-associated bladder SCC and identify predictive biomarkers.

In summary, despite the aforementioned limitations, this case establishes tislelizumab monotherapy as a promising intervention for PD-L1-positive calculus-associated bladder SCC, achieving a durable complete response in a chemotherapy-intolerant patient refusing cystectomy. This response not only supports the potential of immune checkpoint inhibition as an organ-preserving strategy for this rare malignancy but also demonstrates that definitive stone removal can be safely conducted under immunological tumor control, without increasing tumor seeding or metastatic risk. Crucially, these findings advocate for prospective trials to (1): evaluate immunotherapy as first-line therapy for advanced bladder SCC; and (2) define the optimal treatment duration for subtype-specific patient cohort.

## Data Availability

The original contributions presented in the study are included in the article. Further inquiries can be directed to the corresponding authors.
